# Prognostic value of changes in the cardiac arrest rhythms from the prehospital stage to the emergency department in out-of-hospital cardiac arrest patients without prehospital returns of spontaneous circulation: A nationwide observational study

**DOI:** 10.1371/journal.pone.0257883

**Published:** 2021-09-28

**Authors:** Jae Guk Kim, Hyungoo Shin, Jun Hwi Cho, Hyun Young Choi, Wonhee Kim, Jihoon Kim, Shinje Moon, Chiwon Ahn, Juncheol Lee, Youngsuk Cho, Dong Geum Shin, Yoonje Lee

**Affiliations:** 1 Department of Emergency Medicine, Hallym University College of Medicine, Chuncheon, Republic of Korea; 2 Department of Emergency Medicine, Graduate School of Medicine, Kangwon National University, Chuncheon, Republic of Korea; 3 Department of Emergency Medicine, Hanyang University College of Medicine, Hanyang University Guri Hospital, Guri, Republic of Korea; 4 Department of Emergency Medicine, Kangwon National University School of Medicine, Chuncheon, Republic of Korea; 5 Department of Thoracic and Cardiovascular Surgery, Hallym University College of Medicine, Chuncheon, Republic of Korea; 6 Department of Internal Medicine, Hallym University College of Medicine, Seoul, Republic of Korea; 7 Department of Emergency Medicine, Chung-Ang University, College of Medicine, Seoul, Republic of Korea; 8 Department of Emergency Medicine, College of Medicine, Hanyang University, Seoul, Republic of Korea; 9 Department of Emergency Medicine, Kangdong Sacred Heart Hospital, Hallym University College of Medicine, Seoul, Republic of Korea; 10 Department of Cardiology, Kangnam Sacred Heart Hospital, Hallym University Medical Center, Seoul, Republic of Korea; Azienda Ospedaliero Universitaria Careggi, ITALY

## Abstract

**Background:**

This study aimed to assess the prognostic value of the changes in cardiac arrest rhythms from the prehospital stage to the ED (emergency department) in out-of-hospital cardiac arrest (OHCA) patients without prehospital returns of spontaneous circulation (ROSC).

**Methods:**

This retrospective analysis was performed using nationwide population-based OHCA data from South Korea between 2012 and 2016. Patients with OHCA with medical causes and without prehospital ROSC were included and divided into four groups according to the nature of their cardiac arrest rhythms (shockable or non-shockable) in the prehospital stage and in the ED: (1) the shockable and shockable (Shock-Shock) group, (2) the shockable and non-shockable (Shock-NShock) group, (3) the non-shockable and shockable (NShock-Shock) group, and (4) the non-shockable and non-shockable (NShock-NShock) group. The presence of a shockable rhythm was confirmed based on the delivery of an electrical shock. Propensity score matching and multivariate logistic regression analyses were used to assess the effect of changes in the cardiac rhythms on patient outcomes. The primary outcome was sustained ROSC in the ED; the secondary outcomes were survival to hospital discharge and good neurological outcomes at hospital discharge.

**Results:**

After applying the exclusion criteria, 51,060 eligible patients were included in the study (Shock-Shock, 4223; Shock-NShock, 3060; NShock-Shock, 11,509; NShock-NShock, 32,268). The propensity score-matched data were extracted from the six comparative subgroups. For sustained ROSC in the ED, Shock-Shock showed a higher likelihood than Shock-NShock (*P* <0.01) and NShock-NShock (*P* <0.01), Shock-NShock showed a lower likelihood than NShock-Shock (*P* <0.01) and NShock-NShock (*P* <0.01), NShock-Shock showed a higher likelihood NShock-NShock (*P* <0.01). For survival to hospital discharge, Shock-Shock showed a higher likelihood than Shock-NShock (*P* <0.01), NShock-Shock (*P* <0.01), and NShock-NShock (*P* <0.01), Shock-NShock showed a higher likelihood than NShock-Shock (*P* <0.01) and NShock-NShock (*P* <0.01), of sustained ROSC in the ED. For good neurological outcomes, Shock-Shock showed higher likelihood than Shock-NShock (*P* <0.01), NShock-Shock (*P* <0.01), and NShock-NShock (*P* <0.01), Shock-NShock showed better likelihood than NShock-NShock (*P* <0.01), NShock-Shock showed a better likelihood than NShock-NShock (*P* <0.01).

**Conclusion:**

Sustained ROSC in the ED may be expected for patients with shockable rhythms in the ED compared with those with non-shockable rhythms in the ED. For the clinical outcomes, survival to hospital discharge and neurological outcomes, patients with Shock-Shock showed the best outcome, whereas patients with NShock-NShock showed the poorest outcome and Shock-NShock showed a higher likelihood of achieving survival to hospital discharge with no significant differences in the neurological outcomes compared with NShock-Shock.

## Introduction

Adult patients experiencing out-of-hospital cardiac arrest (OHCA) receive numerous medical treatments during resuscitation. Depending on age, comorbidities, and the presumed cause of cardiac arrest, these treatments may contribute to changes in the cardiac arrest rhythms [1].

Cardiac rhythms during a cardiac arrest are categorized as follows: shockable rhythms, such as ventricular fibrillation (VF) and pulseless ventricular tachycardia (VT), and non-shockable rhythms, such as asystoles and pulseless electrical activity (PEA). The cardiac arrest rhythm during resuscitation determines the management of OHCA patients [2]. These rhythms are unstable and change either spontaneously or through interventions such as chest compressions and defibrillations. Temporary returns of spontaneous circulation (ROSC) may also occur, and the presenting cardiac arrest rhythm could thus be a major determinant of outcomes of cardiac arrest patients [3].

Despite advances in cardiopulmonary resuscitation, nearly one-third of the survivors were patients who did not have ROSC in the prehospital setting [4]. Moreover, emergency medical services (EMS) personnel are not allowed to terminate the resuscitation in some countries, including South Korea and Japan, and the application of universal guidelines of the termination of resuscitation is still challenging because of ethical issues. Therefore, several OHCA patients are transported to emergency departments (ED) without prehospital ROSC [4,5].

As the absence of a prehospital ROSC is considered to be an indicator of a poor outcome, the difficulty in predicting the outcome of cardiac arrest patients in the absence of a determinant method, without prehospital ROSC is increasing, [1,6,7]. Prehospital cardiac arrest rhythms, such as shockable rhythms, comprise the most accessible information for ED physicians and can be obtained quickly from the EMS records [6]. The cardiac arrest rhythm in the ED can be changed according to the resuscitation effort in the ED. Therefore, we hypothesized that the cardiac arrest rhythm and its changes can provide physicians with further information to enable the prediction of the prognosis of cardiac arrest patients without prehospital ROSC [8,9].

The relationships between the outcomes of patients and the changes in the cardiac arrest rhythms in OHCA patients have been assessed in several previous studies [1,3,6,8,10–12]. Skogvol et al. reported that cardiac arrest patients with VFs or VTs were more unstable than cardiac arrest patients with PEAs or asystoles, and the patients with VFs or VTs tended to reach sustained ROSC later than patients with PEAs or asystoles [3]. Nordseth et al. showed that increased rates of PEAs to ROSC may improve the overall survival rates of cardiac arrest patients [1]. However, in some of these studies, only OHCA patients with known initial cardiac rhythms were included, and the effect of in-hospital procedures, such as percutaneous coronary interventions (PCIs) and targeted temperature management (TTM), that could influence the outcomes of OHCA patients, were not evaluated [1.3.10]. There have been only a few studies that assessed the effect of changes in the cardiac rhythms from the prehospital stage to the ED admission in OHCA patients without prehospital ROSC. Therefore, this study aimed to assess the prognostic value of changes in cardiac rhythms from the prehospital stage to the ED admission on the outcomes of OHCA patients without prehospital ROSC.

## Materials and methods

### Study design and settings

This was a retrospective observational study using nationwide population-based data from the Out-of-Hospital Cardiac Arrest Surveillance (OHCAS) of the Korean Centers for Disease Control and Prevention (KCDC) between January 2012 to December 2016. The Researcher team applied to the KCDC and received the data in January 2020.

The OHCAS was conducted in the 17 provinces of South Korea (approximately 50 million people) and included detailed patient data. The local ethics committee approved this study in 2020 (Kangnam Sacred Heart Hospital Institutional Review Board; IRB No. 2020-04-013), and the requirement for informed consent was waived because of the retrospective nature of the study and the use of anonymous clinical data for the analysis. The study methodology was consistent with the Strengthening the Reporting of Observational Studies in Epidemiology (STROBE) checklist for observational studies.

### Data source

The OHCAS database is a population-based, emergency medical service (EMS)-assessed OHCA registry, and the retrospective patient cohort that was developed and supported by the Korea Centers for Disease Control and Prevention (CDC) in collaboration with the National Fire Department. The database used in this study comprises national statistics approved by Statistics Korea (Approval Number 117088).

The survey variables were based on the Utstein style guidelines and the Resuscitation Outcome Consortium Project [13,14]. Considering the reality of Korean data collection, some variables have been revised and secured.

Information on the OHCA patients was obtained from the EMS records, and their clinical data, hospital management, and outcomes at hospital discharge were provided by the KCDC. Medical record reviewers from the KCDC visited all the ED and hospitals to which OHCA patients were transported and reviewed the medical records.

OHCAS included basic patient information such as age and sex, places of cardiopulmonary resuscitation (CPR), bystander CPRs, treatments during transportation, survival to hospital discharges, and neurological outcomes at hospital discharges, collected using an appropriately devised survey form.

### Study population

Between January 2012 to December 2016, 142,905 OHCA patients were registered for OHCAS. Among them, adult OHCA patients (≥18 years) without prehospital ROSC were included in the study. The following OHCA patients were excluded from the study: those with prehospital ROSC; cardiac arrest from non-medical or unknown causes; aged <18 years; dead on arrival (DOA) or do-not-resuscitate (DNR) statuses; unknown cardiac rhythms; and invalid or missing data on ROSC, survival, or neurological outcomes due to data loss during medical reviews or transfers to other hospitals.

**Fig 1:**
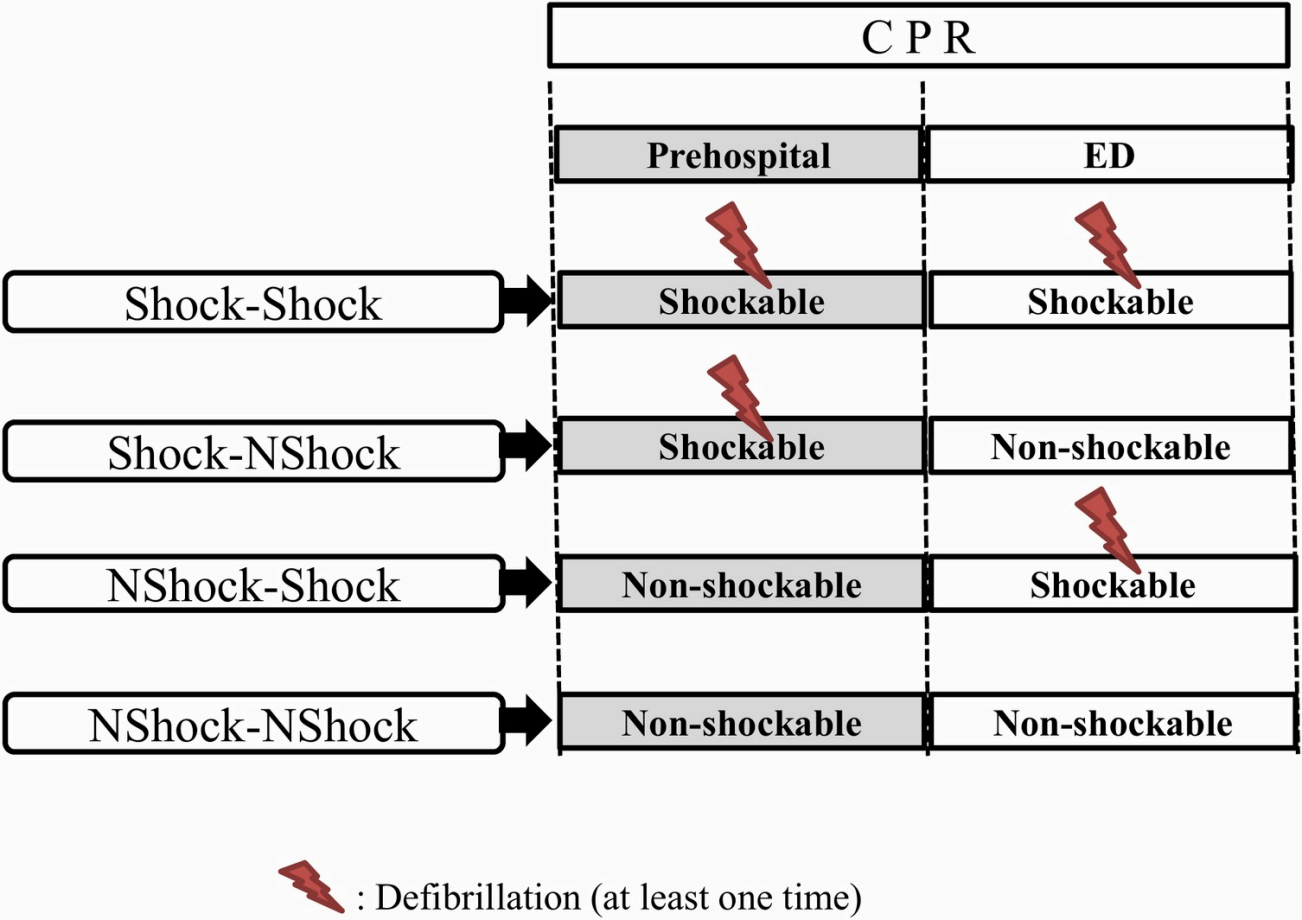
Definitions of the four studied groups (Shock-Shock, Shock-NShock, NShock-Shock, and NShock-NShock) according to the cardiac arrest rhythms (shockable or non-shockable) in the prehospital and emergency department stages. Shock-Shock, shockable and shockable; Shock-NShock, shockable and non-shockable; NShock-Shock, non-shockable and shockable; NShock-NShock, non-shockable and non-shockable; ED, emergency department; CPR, cardiopulmonary resuscitation.

Patients were divided into four groups according to the nature of their prehospital cardiac rhythms, ED cardiac rhythms, and presence of shockable rhythms that were confirmed based on the delivery of an electrical shock: (1) the shockable and shockable rhythm (Shock-Shock) group: patients with shockable rhythms at least once in both the prehospital stage and in the ED, (2) the shockable and non-shockable rhythm (Shock-NShock) group: patients with shockable rhythms at least once in the prehospital stage that converted to a sustained non-shockable rhythm in the ED, (3) the non-shockable and shockable rhythm (NShock-Shock) group: patients with sustained non-shockable rhythms in the prehospital stage that converted to a shockable rhythm at least once in the ED, and (4) the non-shockable and non-shockable rhythm (NShock-NShock) group: patients with sustained non-shockable rhythms in both the prehospital stage and the ED (Fig 1).

The presence of a shockable rhythm was confirmed based on the delivery of an electrical shock.

### Variables

Data on demographic (age, sex), geographical (metropolitan city versus non-metropolitan city), and etiological (cardiac origin versus non-cardiac origin) factors, witnessed cardiac arrest, bystander CPR, CPR locations (public versus non-public), PCI, TTM, mechanical CPR, extracorporeal membrane oxygenation (ECMO), and the time interval from the time of the EMS call to the time of the arrival at the ED, was collected.

Public places were defined as those easily accessible and generally open to people, including roads and commercial facilities. According to the medical records, the cardiac arrest cause was classified as being of cardiac or non-cardiac origin. Conditions such as ischemic heart diseases, arrhythmias, and cardiac tamponades were categorized as being of cardiac origin. The PCIs included angioplasties with balloon and stent insertions. Information on witnessed cardiac arrests (witnessed versus unwitnessed), CPR methods (manual versus mechanical), and the implementation of ECMOs were also obtained from the medical records. A Metropolitan city was defined as one with >1 million people and with first-level administrative divisions, within South Korea. The presence of shockable rhythms in the prehospital stages and the ED were defined as electrical shock delivery by the EMS personnel or an emergency physician. The cardiac rhythm in the prehospital stage was defined as all the prehospital cardiac rhythms encountered by the EMS personnel, from the time of their arrival on the scene and before they arrived at the hospital, during the transportation of the OHCA patients.

Neurological outcomes were evaluated using the Glasgow-Pittsburgh Cerebral Performance Categories (CPC) scale. CPC 1 and 2 indicated good neurological outcomes, and CPC 3–5, poor neurological outcomes. Sustained ROSC in the ED was defined as ROSC for more than 20 min in the ED. The interval (EMS call to ED arrival) was defined as the time from the EMS cardiac arrest call to ED arrival.

### Outcome measures

The primary outcome was a sustained ROSC in the ED; the secondary outcomes were survival to hospital discharge and good neurological outcomes at hospital discharge.

### Statistical analysis

Demographic characteristics according to cardiac arrest rhythms in the prehospital and ED stages were presented as medians and interquartile ranges for the continuous data or frequencies and percentages for the categorical data. The normality of each continuous variable was assessed using the Kolmogorov–Smirnov test. The categorical variables were compared using Pearson’s Chi-square or Fisher’s exact test. The continuous variables were compared using the Kruskal–Wallis tests.

In the primary analysis using multivariate logistic regression, we analyzed the effect of the cardiac rhythm changes on the outcomes. A stepwise backward elimination model was used. Additionally, we tested the effect of the cardiac arrest rhythms on the outcomes of the patients using propensity score-matched samples to minimize the differences in the baseline characteristics between the groups of patients. To compare the two cardiac arrest rhythms, six comparative groups (Shock-Shock vs. Shock-NShock, Shock-Shock vs. NShock-Shock, Shock-NShock vs. NShock-Shock, Shock-Shock vs. NShock-NShock, Shock-NShock vs. NShock-NShock, and NShock-Shock vs. NShock-NShock) were extracted using the propensity score-matched sampling method.

We performed 1:1 (control: treatment = 1:1) propensity score matching to select the participants in both groups. The propensity score was calculated using the multivariate logistic regression model. The matching method used was the nearest neighbor matching. It is a method of matching in the order in which the absolute value of the estimated propensity score difference between the control group and the treatment group is the smallest.

We performed a multivariate logistic regression analysis to determine the prognostic effect of cardiac arrest rhythms on the outcomes of patients for the six propensity score-matched subgroups. The adjusted odds ratios and 95% confidential intervals were calculated from the multivariate logistic regression models.

Variables with *P* <0.05 in the univariate analyses were included in the multivariate regression analysis. All the statistical analyses were performed using the SPSS version 24.0 software (IBM, Armonk, NY, USA) and the R package (R version 3.3.2); *P* <0.05 was considered to be statistically significant.

## Result

### Participant characteristics

Of the 142,905 OHCA patients who were registered during the study period, 91,845 were excluded due to the following reasons: prehospital ROSC (n = 57,576); non-medical or unknown causes of OHCA (n = 27,291); ages lower than 18 years (n = 3064); death on arrival (DOA) or DNR orders (n = 1198); unknown cardiac rhythms (n = 1822); and unknown ROSC, survival, or neurologic outcomes (n = 894).

**Fig 2:**
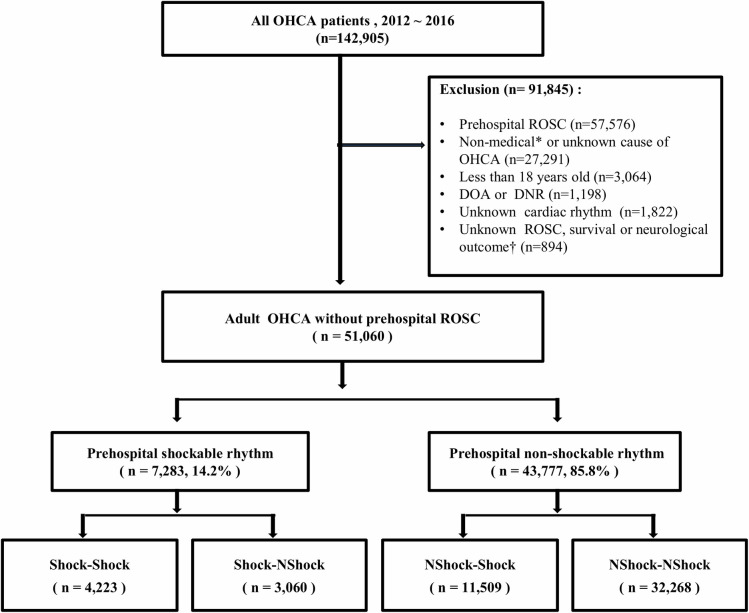
Flow diagram of the studied population. Shock-Shock, shockable and shockable; Shock-NShock, shockable and non-shockable; NShock-Shock, non-shockable and shockable; NShock-NShock, non-shockable and non-shockable; ED, emergency department; OHCA, out-of-hospital cardiac arrest; DOA, dead on arrival; DNR, do not resuscitate; ROSC, return of spontaneous circulation. *non-medical causes included trauma, asphyxia, poisoning, and drowning. †defined as patients with an invalid outcome because of data loss in the medical review or transfer to other hospitals.

The remaining 51,060 OHCA patients were included in the study. Of these, 7283 (14.2%) had shockable rhythms and 43,777 (85.8%) had non-shockable rhythms in the prehospital stage. Among the patients with prehospital shockable rhythms, 4223 (57.9%) maintained their shockable rhythms and 3060 (42.1%) converted to non-shockable rhythms in the ED. Among the patients with initial non-shockable rhythms, 11,509 (26.2%) converted to shockable rhythms and 32,268 (73.8%) maintained their non-shockable rhythms in the ED (Fig 2).

Table 1 summarizes the clinical characteristics of all the patients, of whom 23,774 (46.6%) had sustained ROSC in the ED, 2702 (5.3%) achieved survival at discharge, and 875 (1.7%) were discharged with good neurological outcomes (CPC score of 1 or 2). Age (*P* <0.001); sex (*P* <0.001); metropolitan city (*P* <0.001); the presence of witnessed OHCAs (*P* <0.001); administration of bystander CPRs (*P* <0.001); the places where CPR was administered (*P* <0.001); causes of OHCA (*P* <0.001); application of PCIs (*P* <0.001), TTMs (*P* <0.001), mechanical CPRs (*P* <0.001), ECMOs (*P* <0.001); and the time interval from the time of the EMS call to the time of the arrival at the ED (*P* <0.001) showed significant differences among groups.

**Table 1:** Baseline characteristics and resuscitation variables according to cardiac arrest rhythm in the OHCA patient without ROSC.

Variables	Total (N = 51,060)	Pre-hospital shockable groups		Pre-hospital non-shockable groups		
		Shock-Shock (N = 4,223)	Shock-NShock (N = 3,060)	NShock-Shock (N = 11,509)	NShock-NShock (N = 32,268)	*P-value*
**Age, years**	69 [55–78]	59 [50–71]	63 [52–74]	67 [54–77]	71 [57–79]	< 0.001
**Sex**						
Male	33,442 (65.5%)	3,412 (80.8%)	2,269 (74.2%)	7,859 (68.3%)	19,902 (61.7%)	< 0.001
Female	17,618 (34.5%)	811 (19.2%)	791 (25.8%)	3,650 (31.7%)	12,366 (38.3%)	
**Metropolitan city**	24,492 (48.0%)	2,358 (55.8%)	1,557 (50.9%)	5,220 (45.4%)	15,357 (47.6%)	< 0.001
**Witnessed OHCA**						< 0.001
Yes	27,752 (54.4%)	3,006 (71.2%)	1,987 (64.9%)	6,435 (55.9%)	16,324 (50.6%)	
No	23,308 (45.6%)	1,217 (28.8%)	1,073 (35.1%)	5,074 (44.1%)	15,944 (49.4%)	
**Bystander CPR**	7,889 (15.5%)	1,153 (27.3%)	807 (26.4%)	1,383 (12.0%)	4,546 (14.1%)	< 0.001
**Places of CPR**						< 0.001
Public place	8,755 (17.1%)	1,378 (32.6%)	752 (24.6%)	2,189 (19.0%)	4,436 (13.7%)	
Non-public place	42,305 (82.9%)	2,845 (67.4%)	2,308 (75.4%)	9,320 (81.0%)	27,832 (86.3%)	
**Cause of OHCA**						< 0.001
Cardiac origin	47,124 (92.3%)	4,140 (98.0%)	2,927 (95.7%)	10,790 (93.8%)	29,267 (90.7%)	
Non-cardiac origin	3,936 (7.7%)	83 (2.0%)	133 (4.3%)	719 (6.2%)	3001 (9.3%)	
**PCI**	1,110 (2.2%)	427 (10.1%)	157 (5.1%)	271 (2.4%)	255 (0.8%)	< 0.001
**TTM**	1,924 (3.8%)	369 (8.7%)	267 (8.7%)	328 (2.8%)	960 (3.0%)	< 0.001
**Mechanical CPR**	2,408 (4.7%)	210 (5.0%)	148 (4.8%)	454 (3.9%)	1,596 (4.9%)	< 0.001
**ECMO**	491 (1.0%)	181 (4.3%)	36 (1.2%)	134 (1.2%)	140 (0.4%)	< 0.001
**Time interval, mins**						
EMS call to ED arrival	27.0 [20.0–36.0]	27.0 [20.0–35.0]	30.0 [22.0–40.0]	26.0 [19.0–35.0]	28.0 [20.0–37.0]	< 0.001
**Outcomes**						
**Sustained ROSC in ED**	23,774 (46.6%)	2,234 (52.9%)	1,380 (45.1%)	5,523 (48.0%)	14,637 (45.4%)	< 0.001
**Survival to discharge**	2,702 (5.3%)	601 (14.2%)	308 (10.1%)	496 (4.3%)	1,297 (4.0%)	< 0.001
**Good neurological outcome**	875 (1.7%)	319 (7.6%)	111 (3.6%)	188 (1.6%)	257 (0.8%)	< 0.001

Shock-Shock, shockable rhythm at least one time in both pre-hospital stage and ED; Shock-NShock, shockable rhythm at least one time in the pre-hospital stage but converted and sustained as non-shockable rhythm in ED; NShock-Shock, sustained non-shockable rhythm in the pre-hospital stage but converted shockable rhythm at least one time in ED; NShock-NShock, sustained non-shockable rhythm at both pre-hospital stage and ED; OHCA, out-of-hospital cardiac arrest; ED, emergency department; ROSC, return of spontaneous circulation; CPR, cardiopulmonary resuscitation; TTM, targeted temperature management; PCI, primary coronary intervention; ECMO, extracorporeal membrane oxygenation.

### Outcomes analysis

Significant differences in sustained ROSC in the ED (Shock-Shock, n = 2234 [52.9%] vs. Shock-NShock, n = 1380 [45.1%] vs. NShock-Shock, n = 5523 [48.0%] vs. NShock-NShock, n = 14,637 [45.4%], *P*<0.001), survival to hospital discharge (Shock-Shock, n = 601 [14.2%] vs. Shock-NShock, n = 308 [10.1%] vs. NShock-Shock, n = 496 [4.3%] vs. NShock-NShock, n = 1297 [4.0%], *P*<0.001), and for good neurological outcomes (Shock-Shock, n = 319 [7.6%] vs. Shock-NShock, n = 111 [3.6%] vs. NShock-Shock, n = 188 [1.6%] vs. NShock-NShock, n = 257 [0.8%], **P**<0.001) were noted among the four groups.

### Multivariate logistic analysis of the primary and secondary outcomes in the four cardiac arrest rhythm groups before propensity score-matching

#### Sustained ROSC in the ED

**Fig 3:**
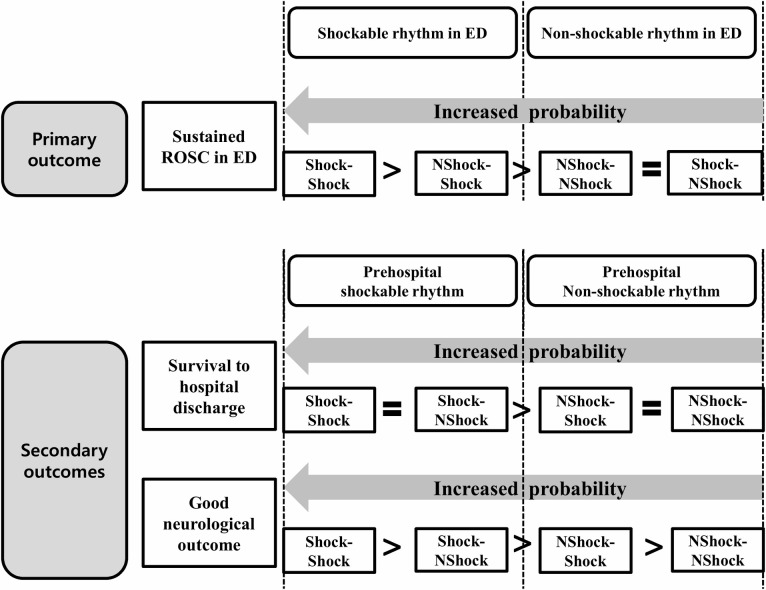
Summarized results of the logistic regression analysis on the effect of changes in the cardiac rhythms from the prehospital to the emergency department stages on the outcomes of OHCA patients without prehospital ROSC before propensity score matching. Shock-Shock, shockable and shockable; Shock-NShock, shockable and non-shockable; NShock-Shock, non-shockable and shockable; NShock-NShock, non-shockable and non-shockable; ROSC, return of spontaneous circulation; ED, emergency department.

The two groups with shockable rhythms in the ED (Shock-Shock and NShock-Shock) had higher likelihoods of sustained ROSC in the ED than the other groups (Shock-NShock and NShock-NShock) (*P* <0.01). Shock-Shock was associated significantly with a higher likelihood of sustained ROSC in the ED than NShock-Shock (adjusted odds ratio [AOR][95% confidence interval [CI]], 1.34 [1.22–1.48], P<0.01); however, no significant differences were observed between the Shock-NShock and NShock-NShock groups (AOR [95% CI], 0.93 [0.86–1.01], *P* = 0.93) (Table 2 and Fig 3).

**Table 2:** Multivariate logistic regression for survival to hospital discharge and good neurological outcome at hospital discharge.

Outcome	Groups	AOR	95%CI		*P*	AOR	95%CI		*P*	AOR	95%CI		*P*
Sustained ROSC in ED†	Shock-Shock	**1.34**	**1.22**	**1.48**	**<0.01**	**1.12**	**1.04**	**1.21**	**<0.01**	**1.25**	**1.17**	**1.34**	**<0.01**
	Shock-NShock	1.00				**0.83**	**0.77**	**0.90**	**<0.01**	0.93	0.86	1.01	0.89
	NShock-Shock	**1.19**	**1.10**	**1.29**	**<0.01**	1.00				**1.11**	**1.06**	**1.16**	**<0.01**
	NShock-NShock	**1.06**	**0.99**	**1.15**	**0.89**	**0.89**	**0.85**	**0.93**	**<0.01**	1.00			
Survival to hospital discharge*	Shock-Shock	1.19	0.96	1.47	0.10	**2.46**	**2.05**	**2.96**	**<0.01**	**2.51**	**2.14**	**2.95**	**<0.01**
	Shock-NShock	1.00				**2.06**	**1.67**	**2.55**	**<0.01**	**2.10**	**1.74**	**2.54**	**<0.01**
	NShock-Shock	**0.48**	**0.39**	**0.59**	**<0.01**	1.00				1.02	0.87	1.18	0.78
	NShock-NShock	**0.47**	**0.39**	**0.57**	**<0.01**	0.97	0.84	1.13	0.78	1.00			
Good neurological outcome*	Shock-Shock	**1.70**	**1.26**	**2.30**	**<0.01**	**2.40**	**1.87**	**3.08**	**<0.01**	**3.28**	**2.60**	**4.14**	**<0.01**
	Shock-NShock	1.00				**1.41**	**1.02**	**1.95**	**<0.01**	**1.92**	**1.41**	**2.62**	**<0.01**
	NShock-Shock	**0.70**	**0.51**	**0.98**	**<0.01**	1.00				**1.36**	**1.06**	**1.75**	**0.01**
	NShock-NShock	**0.51**	**0.38**	**0.70**	**<0.01**	**0.73**	**0.57**	**0.94**	**0.01**	**1.00**			

Shock-Shock, shockable rhythm at least one time in both pre-hospital stage and ED; Shock-NShock, shockable rhythm at least one time in the pre-hospital stage but converted and sustained as non-shockable rhythm in ED; NShock-Shock, sustained non-shockable rhythm in the pre-hospital stage but converted shockable rhythm at least one time in ED; NShock-NShock, sustained non-shockable rhythm at both pre-hospital stage and ED; ROSC, return of spontaneous circulation; CPR, cardiopulmonary resuscitation; AOR, adjusted odds ratio; CI, confidence interval.

†Adjusted odds ratio for age, sex, metropolitan city, witnessed out-of-hospital cardiac arrest, bystander CPR, places of CPR, cause of OHCA.

*Adjusted odds ratio for age, sex, metropolitan city, witnessed out-of-hospital cardiac arrest, bystander CPR, places of CPR, cause of OHCA, hospital ROSC, primary coronary intervention, targeted temperature management, mechanical CPR, extracorporeal membrane oxygenation, and time interval from EMS call to ED arrival.

#### Survival to hospital discharge

Both the prehospital shockable rhythm groups (Shock-Shock and Shock-NShock) had higher likelihoods of survival to hospital discharge than the prehospital non-shockable rhythm groups (NShock-Shock and NShock-NShock) (*P* <0.01). No significant differences were noted in this variable between the Shock-Shock and Shock-NShock groups (adjusted OR [95% CI], 1.19 [0.96–1.47], P = 0.10) and between the NShock-Shock and NShock-NShock groups (AOR [95% CI], 1.02 [0.87–1.18], P = 0.78) (Table 2 and Fig 3).

#### Good neurological outcomes

Both the prehospital shockable rhythm groups had higher likelihoods of good neurological recoveries than the prehospital non-shockable rhythm groups (*P* <0.01). Shock-Shock was associated significantly with a higher likelihood of good neurological outcomes than Shock-NShock (AOR [95% CI], 1.70 [1.26–2.30], P<0.01), and NShock-Shock was associated significantly with a higher likelihood of good neurological outcomes than NShock-NShock (AOR [95% CI], 1.36 [1.06–1.75], *P* = 0.01) (Table 2 and Fig 3).

The presence of shockable rhythm in the ED was significantly associated with a higher likelihood of good neurological outcomes but did not affect survival to hospital discharge. Moreover, it was associated with an increased likelihood of sustained ROSC, irrespective of the prehospital cardiac arrest rhythm.

### Multivariate logistic analysis of the primary and secondary outcomes in the four cardiac arrest rhythm groups after propensity score-matching

The propensity score-matched data were extracted from the six comparative subgroups. The comparison of Shock-Shock vs. Shock-NShock (each n = 3,060), Shock-Shock vs. NShock-Shock (each n = 4,223), Shock-NShock vs. NShock-Shock (each n = 3,060), Shock-Shock vs. NShock-NShock (each n = 4,223), Shock-NShock vs. NShock-NShock (each n = 3,060), and NShock-Shock vs. NShock-NShock (each n = 11,509). (S1 Table)

#### Sustained ROSC in the ED

**Fig 4:**
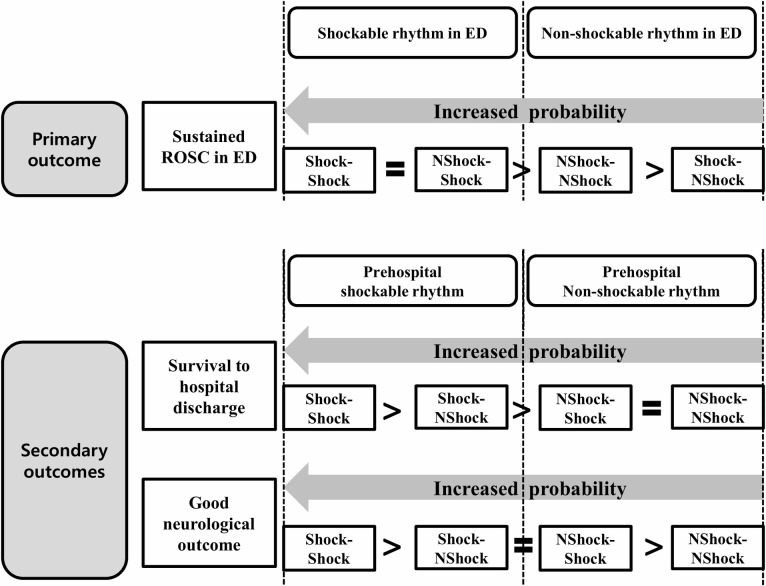
Summarized results of the logistic regression analysis on the effect of changes in the cardiac rhythms from the prehospital to emergency department stages on the outcomes of the OHCA patients without prehospital ROSC after propensity score matching. Shock-Shock, shockable and shockable; Shock-NShock, shockable and non-shockable; NShock-Shock, non-shockable and shockable; NShock-NShock, non-shockable and non-shockable; ROSC, return of spontaneous circulation; ED, emergency department.

The two groups with the shockable rhythm in the ED (Shock-Shock and NShock-Shock) had a higher likelihood of sustained ROSC in the ED than the other groups (NShock-NShock and Shock-NShock) (*P* <0.01). Shock-NShock was associated significantly with a lower likelihood of sustained ROSC in the ED than NShock-NShock (AOR [95% CI], 0.79 [0.71–0.87], *P* <0.01); however, no significant differences were observed between the Shock-Shock and NShock-Shock groups (AOR [95% CI], 1.03 [0.95–1.13], *P* = 0.4) (Table 3 and Fig 4).

**Table 3:** Multivariable logistic regression analysis for the propensity score-matched sample between four groups for outcomes.

**Outcomes**	**Groups**	**Shock-Shock vs. Shock-NShock (Ref.)**			**Shock-Shock vs. NShock-Shock (Ref.)**			**Shock-NShock vs. NShock-Shock (Ref.)**		
		AOR	95% CI	*P*	AOR	95% CI	*P*	AOR	95% CI	*P*
Sustained ROSC in ED†	Reference	1.00			1.00			1.00		
	Test	**1.28**	**1.16–1.42**	**<0.01**	1.03	0.95–1.13	0.4	**0.77**	**0.70–0.86**	**<0.01**
Survival to discharge*	Reference	1.00			1.00			1.00		
	Test	**1.31**	**1.09–1.58**	**<0.01**	**1.76**	**1.49–2.07**	**<0.01**	**1.85**	**1.28–1.95**	**<0.01**
Good neurological outcome*	Reference	1.00			1.00			1.00		
	Test	**1.71**	**1.31–2.24**	**<0.01**	**1.99**	**1.58–2.49**	**<0.01**	1.14	0.84–1.54	0.39
**Outcomes**	**Groups**	**Shock-Shock vs. NShock-NShock (Ref.)**			**Shock-NShock vs. NShock-NShock (Ref.)**			**NShock-Shock vs. NShock-NShock (Ref.)**		
		AOR*	95% CI	*P*	AOR*	95% CI	*P*	AOR*	95% CI	*P*
Sustained ROSC in ED†	Reference	1.00			1.00			1.00		
	Test	**1.15**	**1.05–1.25**	**<0.01**	**0.79**	**0.71–0.87**	**<0.01**	**1.13**	**1.07–1.19**	**<0.01**
Survival to discharge*	Reference	1.00			1.00			1.00		
	Test	**1.93**	**1.64–2.27**	**<0.01**	**1.41**	**1.16–1.72**	**<0.01**	0.91	0.79–1.04	0.18
Good neurological outcome*	Reference	1.00			1.00			1.00		
	Test	**3.93**	**2.98–5.17**	**<0.01**	**2.17**	**1.53–3.08**	**<0.01**	**1.55**	**1.21–1.99**	**<0.01**

Shock-Shock, shockable rhythm at least one time in both pre-hospital stage and ED; Shock-NShock, shockable rhythm at least one time in the pre-hospital stage but converted and sustained as non-shockable rhythm in ED; NShock-Shock, sustained non-shockable rhythm in the pre-hospital stage but converted shockable rhythm at least one time in ED; NShock-NShock, sustained non-shockable rhythm at both pre-hospital stage and ED; ROSC, return of spontaneous circulation; CPR, cardiopulmonary resuscitation; AOR, adjusted odds ratio; CI, confidence interval.

†Adjusted odds ratio for age, sex, metropolitan city, witnessed out-of-hospital cardiac arrest, bystander CPR, places of CPR, cause of OHCA.

*Adjusted odds ratio for age, sex, metropolitan city, witnessed out-of-hospital cardiac arrest, bystander CPR, places of CPR, cause of OHCA, hospital ROSC, primary coronary intervention, targeted temperature management, mechanical CPR, extracorporeal membrane oxygenation, and time interval from EMS call to ED arrival.

#### Survival to hospital discharge

Shock-Shock showed a higher survival to hospital discharge than Shock-NShock (AOR [95% CI],1.31[1.09–1.58], *P* <0.01), NShock-Shock (AOR [95% CI],1.76[1.49–2.07], *P* <0.01), and NShock-NShock (AOR [95% CI],1.93[1.64–2.27], *P* <0.01). Shock-NShock showed higher survival to hospital discharge than NShock-Shock (AOR [95% CI],1.85[1.28–1.95], *P* <0.01) and NShock-NShock (AOR [95% CI],1.41[1.16–1.72], *P* <0.01). But there was no significant difference was observed between NShock-Shock and NShock-NShock groups (AOR [95% CI],0.91[0.79–1.04], *P* = 0.18) (Table 3 and Fig 4).

#### Good neurological outcomes

Shock-Shock showed better neurological recovery than Shock-NShock (AOR [95% CI], 1.71[1.31–2.24], *P* <0.01), NShock-Shock (AOR [95% CI], 1.99[1.58–2.49], *P* <0.01), and NShock-NShock (AOR [95% CI], 3.93[2.98–5.17], *P* <0.01). Shock-NShock showed better neurological recovery than NShock-NShock (AOR [95% CI], 2.17[1.53–3.08], *P* <0.01). NShock-Shock showed better neurological recovery than NShock-NShock (AOR [95% CI], 1.55[1.21–1.99], *P*<0.01); however, no significant difference were observed between the Shock-NShock and NShock-Shock groups (AOR [95% CI], 1.14 [0.84–1.54], *P* = 0.39). (Table 3 and Fig 4).

## Discussion

In OHCA patients without prehospital ROSC, shockable rhythms in the ED could increase the rate of sustained ROSC in the ED regardless of the prehospital cardiac arrest rhythms. For the clinical outcomes, survival to hospital discharge, and the neurological outcomes, Shock-Shock was associated with the best outcome for OHCA patients than the other groups and Shock-NShock showed a higher survival to hospital discharge but no significant differences in the neurological outcome compared with NShock-Shock. Among the four groups of cardiac arrest rhythms, NShock-NShock may have the poorest outcome.

Several studies have examined the effect of changes in cardiac arrest rhythms during resuscitation [1–3,6,8,9,11]. However, some studies reported only the effect of conversion to shockable rhythms in cardiac arrest patients with initial non-shockable rhythms [6,8,11], while others evaluated mainly the associations of transitions between cardiac rhythms with ROSC [1–3,9]. Clinical outcomes such as survival and neurological outcomes were evaluated to a limited extent in these studies [1–3,9]. Moreover, there has been no research involving OHCA patients without prehospital ROSC among these studies. Therefore, we aimed to investigate the effect of changes in cardiac rhythms on the outcomes of OHCA patients without prehospital ROSC.

In the present study, Shock-Shock showed a higher likelihood of survival to hospital discharge and good neurological recovery compared with the other groups, and Shock-NShock showed a higher likelihood in survival to hospital discharge, although not for good neurological outcomes compared with NShock-Shock.

OHCA patients with shockable rhythms were reported to have increased survival rates and better neurological outcomes than patients with non-shockable rhythms [13,15,16]. However, in this study, Shock-NShock did not show better neurological outcomes than NShock-Shock. This difference may have been because this study only included OHCA patients without prehospital ROSC with relatively long CPR durations to sustained ROSC. Considering that a major cause of poor outcomes after cardiac arrest is hypoxic-ischemic brain injuries and with cerebral blood flow being extremely low during CPR following prolonged cardiac arrests [17–20], a prolonged CPR duration could have been the cause of the poor neurological outcomes of the patients.

Our study showed that shockable rhythms in the ED were associated with better neurological outcomes (Shock-Shock vs. Shock-NShock and NShock-Shock vs. NShock-NShock) and survival to hospital discharge (Shock-Shock vs. Shock-NShock).

OHCA patients without prehospital ROSC remain vulnerable even after ROSC in the hospital, as they tend to have high degrees of brain damage and could die relatively early after arrival at the hospital [21]. In this study, the time intervals from cardiac arrest to sustained ROSC in the ED were not evaluated because reliable data were unavailable. However, considering the benefits of defibrillation and the higher rates of ROSC in patients with shockable rhythms [22,23], patients with shockable rhythm in the ED (Shock-Shock and NShock-Shock) may experience shorter delays to sustained ROSC in the ED after cardiac arrest than patients with non-shockable rhythms in the ED (Shock-NShock and Nshock-NShock). Therefore, OHCA patients with shockable rhythms in the ED could experience less severe hypoxic brain injuries than the groups with non-shockable rhythms in the ED, which could contribute to the higher rates of survival to hospital discharge and good neurological outcomes.

The findings of this study showed that shockable rhythms in the ED were also associated with higher rates of sustained ROSC in the ED than prehospital shockable rhythms. This may have been due to the benefits of shockable rhythms, which translates to ROSC after defibrillations. However, considering that advanced life support is not provided in prehospital settings by the Korean EMS, this result could also be due to the use of epinephrine in the ED. In several previous studies, the effect of adrenaline in cardiac arrest patients was examined [2,24,25]. Although none of these studies could demonstrate improved survival to hospital discharge, they reported increased rates of ROSC after adrenaline administration. In another study, the use of intravenous adrenaline increased the rates of transition from non-shockable to shockable rhythms [2]. However, because reliable data on adrenaline use was unavailable, this additional analysis was not performed in the present study. Therefore, further studies are required to elucidate the main factors that contribute to sustained ROSC in the ED for OHCA patients without prehospital ROSC.

During post-cardiac arrest care, the rate of TTM of this study was relatively low. The exact reasons for the low TTM ratio are not identifiable; however the possible causes for the estimation are as follows: not all of the hospitals’ EMS personnel that transport the cardiac arrest patients have TTM devices and the relevant medical staff, and university hospitals that do have TTM devices and medical staff have a limited number of the equipment. In addition, TTM is one of the most expensive medical procedures in South Korea; therefore, considering the additional cost of treatment such as intensive care unit stays, this was not used in all the patients due to family rejections arising from financial difficulties. Considering the neuroprotective effects of TTM, this could have been the reason for the different results of other studies with a high rate of TTM.

The results of this study indicate the importance of the resuscitation efforts in the prehospital stage and in the ED aimed at obtaining and maintaining shockable rhythms, even for OHCA patients without prehospital ROSC, to attain for them to obtain a better chance of good survival and neurological outcomes. Therefore, the continuation of resuscitation effort to convert to shockable rhythm from non-shockable rhythm and maintain the shockable rhythm should be considered even for OHCA patients without prehospital ROSC. The results are expected to be particularly useful for ED physicians who manage OHCA patients with prehospital non-shockable rhythms, especially since these patients comprise the majority of OHCA patients without prehospital ROSC.

Nevertheless, our study had some limitations, which should be noted. First, because this was a retrospective observational study, the possibility of reporting bias and selection bias cannot be ruled out. Therefore, the results should be interpreted cautiously. Second, data on cardiac rhythm in the prehospital stage were based on the rhythms recorded by the EMS personnel, which could have been different from the true cardiac rhythms after the sudden collapse of the patient. Third, long-term outcomes after hospital discharge were not evaluated [26]. Therefore, the rates of survival and good neurological outcomes in this study could have been different from those of other studies that evaluated the long-term outcomes of OHCA patients. Fourth, considering the diversity in medical systems and medical resources including the EMS and resuscitation protocols in several countries, these results cannot be generalized to other countries; the results of similar studies in other countries could differ from those of this study. However, we used a standardized Utstein template for the data collection and adjusted for hospital variances in all the analytical models in the present study, thus circumventing some of these problems. Finally, we did not assess the effect of the type of non-shockable rhythms on the patient’s outcomes. However, recent two studies reported that patients with shockable rhythm conversions from initial non-shockable rhythms showed better outcomes than those without conversions, regardless of the type of non-shockable rhythm (initial PEA or asystole) [6,27]. Therefore, the effect of the type of initial non-shockable rhythms (PEA or asystole) on the result of this study though to be minimal.

## Conclusions

Sustained ROSC in the ED may be expected for patients with shockable rhythms in ED compared with those with non-shockable rhythms in the ED. In terms of survival to hospital discharge and neurological outcomes, patients with Shock-Shock showed the best outcome, whereas patients with NShock-NShock showed the poorest outcome while Shock-NShock showed a higher likelihood of achieving survival to hospital discharge, although with no significant difference in the neurological outcome compared with NShock-Shock. Therefore, resuscitation efforts aimed at obtaining and maintaining shockable rhythms in the prehospital stage and the ED should be considered to attain better outcomes for OHCA patients without prehospital ROSC.

## Supporting information

S1 Table Demographics between four groups according to cardiac arrest rhythms in propensity score-matched sample. Shock-Shock, shockable rhythm at least one time in both pre-hospital stage and ED; Shock-NShock, shockable rhythm at least one time in the pre-hospital stage but converted and sustained as non-shockable rhythm in ED; NShock-Shock, sustained non-shockable rhythm in the pre-hospital stage but converted shockable rhythm at least one time in ED; NShock-NShock, sustained non-shockable rhythm at both pre-hospital stage and ED; OHCA, out-of-hospital cardiac arrest; ED, emergency department; ROSC, the return of spontaneous circulation; CPR, cardiopulmonary resuscitation; TTM, targeted temperature management; PCI, primary coronary intervention; ECMO, extracorporeal membrane oxygenation.
(PDF)

S1 Dataset Minimal underlying data. (ZIP)
