# Supplementary material for: Prognostic value of changes in the cardiac arrest rhythms from the prehospital stage to the emergency department in out-of-hospital cardiac arrest patients without prehospital returns of spontaneous circulation: A nationwide observational study
Source: PLoS One. 2021 Sep 28;16(9):e0257883. doi: 10.1371/journal.pone.0257883 (PMC8478240; doi:10.1371/journal.pone.0257883)
Supplement: S1 Table — Shock-Shock, shockable rhythm at least one time in both pre-hospital stage and ED; Shock-NShock, shockable rhythm at least one time in the pre-hospital stage but converted and sustained as non-shockable rhythm in ED; NShock-Shock, sustained non-shockable rhythm in the pre-hospital stage but converted shockable rhythm at least one time in ED; NShock-NShock, sustained non-shockable rhythm at both pre-hospital stage and ED; OHCA, out-of-hospital cardiac arrest; ED, emergency department; ROSC, the return of spontaneous circulation; CPR, cardiopulmonary resuscitation; TTM, targeted temperature management; PCI, primary coronary intervention; ECMO, extracorporeal membrane oxygenation. (PDF) [file pone.0257883.s001.pdf]

S1 Table. Demographics between four groups according to cardiac arrest rhythms in propensity score-matched sample

Variables	Shock-Shock vs. Shock-NShock			Shock-Shock vs. NShock-Shock			Shock-NShock vs. NShock-Shock		
	Shock-Shock (N=3060)	Shock-NShock (N=3060)	*P*	NShock-Shock (N=4223)	Shock-Shock (N=4223)	*P*	NShock-Shock (N=3060)	Shock-NShock (N=3060)	*P*
**Age, years**	63[53-73]	63 [52-74]	0.111	59[50-71]	59 [50-71]	0.826	63[52-75]	63 [52-74]	0.736
**Sex**			**0.009**			0.978			0.519
Female	702 (22.9%)	791 (25.8%)		813 (19.3%)	811 (19.2%)		768 (25.1%)	791 (25.8%)	
Male	2358 (77.1%)	2269 (74.2%)		3410 (80.7%)	3412 (80.8%)		2292 (74.9%)	2269 (74.2%)	
**Metropolitan city**	1559 (50.9%)	1557 (50.9%)	0.980	2262 (53.6%)	2358 (55.8%)	**0.038**	1577 (51.5%)	1557 (50.9%)	0.627
**Witnessed OHCA**			0.215			0.701			0.468
No	1026 (33.5%)	1073 (35.1%)		1234 (29.2%)	1217 (28.8%)		1045 (34.2%)	1073 (35.1%)	
Yes	2034 (66.5%)	1987 (64.9%)		2989 (70.8%)	3006 (71.2%)		2015 (65.8%)	1987 (64.9%)	
**Bystander CPR**	799 (26.1%)	807 (26.4%)	0.839	988 (23.4%)	1153 (27.3%)	**< 0.001**	819 (26.8%)	807 (26.4%)	0.750
**Places of CPR**			0.443			0.263			0.677
Non-public place	2281 (74.5%)	2308 (75.4%)		2894 (68.5%)	2845 (67.4%)		2323 (75.9%)	2308 (75.4%)	
Public place	779 (25.5%)	752 (24.6%)		1329 (31.5%)	1378 (32.6%)		737 (24.1%)	752 (24.6%)	
**Cause of OHCA**			**0.001**			0.574			0.800
Non-cardiac origin	82 (2.7%)	133 (4.3%)		75 (1.8%)	83 (2.0%)		128 (4.2%)	133 (4.3%)	
Cardiac origin	2978 (97.3%)	2927 (95.7%)		4148 (98.2%)	4140 (98.0%)		2932 (95.8%)	2927 (95.7%)	
**PCI**	151 (4.9%)	157 (5.1%)	0.770	260 (6.2%)	427 (10.1%)	**< 0.001**	137 (4.5%)	157 (5.1%)	0.256
**TTM**	256 (8.4%)	267 (8.7%)	0.647	244 (5.8%)	369 (8.7%)	**< 0.001**	226 (7.4%)	267 (8.7%)	0.060
**Mechanical CPR**	132 (4.3%)	148 (4.8%)	0.359	178 (4.2%)	210 (5.0%)	0.107	128 (4.2%)	148 (4.8%)	0.242
**ECMO**	34 (1.1%)	36 (1.2%)	0.904	112 (2.7%)	181 (4.3%)	**< 0.001**	34 (1.1%)	36 (1.2%)	0.904
**Time interval, mins**									
EMS call to ED arrival	27 [20-35]	30 [22-40]	<0.001	26 [19-34]	27 [20-35]	0.002	26 [20-34]	30 [22-40]	<0.001
**Outcomes**									
**Sustained ROSC in ED**	1556 (50.8%)	1380 (45.1%)	**< 0.001**	2188 (51.8%)	2234 (52.9%)	0.327	1569 (51.3%)	1380 (45.1%)	**< 0.001**
**Survival to discharge**	367 (12.0%)	308 (10.1%)	**0.018**	324 (7.7%)	601 (14.2%)	**< 0.001**	210 (6.9%)	308 (10.1%)	**< 0.001**
**Good neurological outcome**	172 (5.6%)	111 (3.6%)	**< 0.001**	138 (3.3%)	319 (7.6%)	**< 0.001**	95 (3.1%)	111 (3.6%)	0.288

Variables	Shock-Shock vs. NShock-NShock			Shock-NShock vs. NShock-NShock			NShock-Shock vs. NShock-NShock		
	NShock-NShock (N=4223)	Shock-Shock (N=4223)	*P*	NShock-NShock (N=3060)	Shock-NShock (N=3060)	*P*	NShock-NShock (N=11509)	NShock-Shock (N=11509)	*P*
**Age, years**	59 [50-71]	59 [50-71]	0.121	63 [52-75]	63 [52-74]	0.799	67[54-77]	67 [54-77]	0.469
**Sex**			0.912			0.598			0.454
Female	816 (19.3%)	811 (19.2%)		772 (25.2%)	791 (25.8%)		3704 (32.2%)	3650(31.7%)	
Male	3407(80.7%)	3412 (80.8%)		2288 (74.8%)	2269(74.2%)		7805 (67.8%)	7859(68.3%)	
**Metropolitan city**	2266(53.7%)	2358 (55.8%)	**0.047**	1556 (50.8%)	1557(50.9%)	1.000	5199 (45.2%)	5220(45.4%)	0.791
**Witnessed OHCA**			0.962						0.623
No	1220(28.9%)	1217 (28.8%)		1058 (34.6%)	1073(35.1%)		5112 (44.4%)	5074(44.1%)	
Yes	3003(71.1%)	3006 (71.2%)		2002 (65.4%)	1987(64.9%)		6397 (55.6%)	6435(55.9%)	
**Bystander CPR**	1097(26.0%)	1153 (27.3%)	0.176	800 (26.1%)	807 (26.4%)	0.862	1281 (11.1%)	1383(12.0%)	**0.037**
**Places of CPR**			0.871			0.420			0.132
Non-public place	2853(67.6%)	2845 (67.4%)		2336 (76.3%)	2308(75.4%)		9410 (81.8%)	9320(81.0%)	
Public place	1370(32.4%)	1378 (32.6%)		724 (23.7%)	752 (24.6%)		2099 (18.2%)	2189(19.0%)	
**Cause of OHCA**			0.690			0.270			0.246
Non-cardiac origin	77 (1.8%)	83 (2.0%)		115 (3.8%)	133 (4.3%)		676 (5.9%)	719 (6.2%)	
Cardiac origin	4146(98.2%)	4140 (98.0%)		2945 (96.2%)	2927(95.7%)		10833(94.1%)	10790(93.8%)	
**PCI**	221 (5.2%)	427 (10.1%)	**< 0.001**	138 (4.5%)	157 (5.1%)	0.283	231 (2.0%)	271 (2.4%)	0.078
**TTM**	286 (6.8%)	369 (8.7%)	**0.001**	230 (7.5%)	267 (8.7%)	0.092	239 (2.1%)	328 (2.8%)	**< 0.001**
**Mechanical CPR**	170 (4.0%)	210 (5.0%)	**0.041**	132 (4.3%)	148 (4.8%)	0.359	411 (3.6%)	454 (3.9%)	0.145
**ECMO**	96 (2.3%)	181 (4.3%)	**< 0.001**	28 (0.9%)	36 (1.2%)	0.379	104 (0.9%)	134 (1.2%)	0.059
**Time interval, mins**									
EMS call to ED arrival	27 [20-37]	27 [20-35]	0.331	28 [20-36]	30 [22-40]	<0.001	28 [20-37]	26 [19-35]	<0.001
**Outcomes**									
**Sustained ROSC in ED**	2085(49.4%)	2234 (52.9%)	**0.002**	1545 (50.5%)	1380 45.1%)	**< 0.001**	5164 (44.9%)	5523 (48.0%)	**< 0.001**
**Survival to discharge**	304 (7.2%)	601 (14.2%)	**< 0.001**	223 (7.3%)	308 (10.1%)	**< 0.001**	494 (4.3%)	496 (4.3%)	0.974
**Good neurological outcome**	72 (1.7%)	319 (7.6%)	**< 0.001**	52 (1.7%)	111 (3.6%)	**< 0.001**	112 (1.0%)	188 (1.6%)	**< 0.001**

Shock-Shock, shockable rhythm at least one time in both pre-hospital stage and ED; Shock-NShock, shockable rhythm at least one time in the pre-hospital stage but converted and sustained as non-shockable rhythm in ED; NShock-Shock, sustained non-shockable rhythm in the pre-hospital stage but converted shockable rhythm at least one time in ED; NShock-NShock, sustained non-shockable rhythm at both pre-hospital stage and ED; OHCA, out-of-hospital cardiac arrest; ED, emergency department; ROSC, the return of spontaneous circulation; CPR, cardiopulmonary resuscitation; TTM, targeted temperature management; PCI, primary coronary intervention; ECMO, extracorporeal membrane oxygenation

1
